# A Compliment to the Physician

**Published:** 1879-12

**Authors:** 


A Beautiful Compliment to the Physician.—I dare not place any gift, however beautiful, or any success however brilliant, above the talent or the skill which can relieve a single pang, and the self-devotion which lays them at the feet of the humblest fellow creature.—*Oliver Wendell Holmes.*

EDUCATION is literally "a leading from;" it may be from ignorance to knowledge; from vice to virtue. This is education in a good sense. But we may be educated, "led from" virtue to vice; from truth to error; so that in its largest acceptation, education is the being "led from" an old state or habit of thought, feeling, and action, into some other state of thought, feeling, and action. But we may educate the body as well as the mind and heart; we may educate it from a sickly, diseased condition into robust health and manly vigor; we may also bring it from these down to rottenness and imbecility. A true and comprehensive education, in reference especially to our children, is that which leads out the bodily powers to greater strength; the mental to greater activities; the moral to greater purity, beneficence, and nobility—all these should be done at the same time, although in different degrees.

